# Musculoskeletal disorders increases the insomnia severity in nurses

**DOI:** 10.5935/1984-0063.20200115

**Published:** 2022

**Authors:** Remziye Cici, Gülay Yilmazel

**Affiliations:** Hitit University Faculty of Health Sciences, Nursing - Çorum - Çorum - Turkey.

**Keywords:** Musculoskeletal Diseases, Nurses, Sleep

## Abstract

**Objectives:**

To determine prevalence of musculoskeletal disorders and the relationship between insomnia severity among nurses working in a training and research hospital.

**Material and Methods:**

This descriptive study was carried out with 293 nurses. Cornell musculoskeletal disorders questionnaire for musculoskeletal disorders and insomnia severity index were used to determine the insomnia severity.

**Results:**

In our study, the severity of the discomfort was mostly moderate and mild in painful areas. There was a significant difference in terms of gender, educational status, marital status, seniority, types of working, and presence of chronic diseases for musculoskeletal disorders in different body regions (p<0.05). It was determined that the threshold level of insomnia was significantly higher in those with musculoskeletal disorders in other body regions except the knee and lower leg region (p<0.05).

**Conclusion:**

In our study, it was determined that musculoskeletal disorders were common in the upper body areas and the severity of insomnia was at the threshold level. Neck, right shoulder and right upper arm were risky body areas for insomnia.

## INTRODUCTION

Musculoskeletal disorders (MSD) defined with two components as “muscular system and the skeletal system”, which are acute or chronically affecting disorders that triggered by work may impair the function of different body parts depending on the workplace and the employee^1^.

Musculoskeletal disorders are one of the most important challenges of occupational health in today’s world and are present in many jobs^2,3^.

Stress experienced in the work environment with many triggering factors such as movements that force the posture, traumas caused by repetitive movements, intense physical workload, disproportionate or improper use of body parts creates a serious load on the musculoskeletal system^4^. Work-related MSD have been reported to be highly prevalent in developing and developed countries^5,6,7^.

Although MSD are high in the general population, it is seen that especially those working in the field of medical care face these problems more^8,9^. Assessment of the incidence of MSD in healthcare providers is seriously addressed in many countries^10^. Among those who direct contact with patients, especially nurses among the health professionals are under the risk of MSD^11,12^. It has been reported that the prevalence of MSD in nurses worldwide is between 40-90%^13^.

Musculoskeletal disorders are associated with improper body mechanics^14^ as well as poor sleep quality in health workers^15^. Sleep problems occur as a result of irregular working hours that do not comply with routine sleep-wake hours and stress in the work environment^16^. In a study conducted with hospital staff (58% of 3,593 participants were nurses), 57% of the participants were reported to have poor sleep^15^. Sleep problems may lead to decreased work performance and attention, chronic fatigue, mood disorders, gastrointestinal system problems, increased risk of hypertension and cardiovascular disease^16^, and pain sensitivity^15^. However, the link between pain and insomnia may not be determined exactly^15^.

The aim of study was to determine prevalence of the musculoskeletal disorders and the relationship between insomnia severity among nurses working in a training and research hospital.

## MATERIAL AND METHODS

This descriptive study was carried out between October-November 2020 in a city located Black Sea Region of Turkey. The population of study was consisted of 435 nurses working in an education and research hospital. For the study, all surgical and internal departments were chosen in hospital and 293 nurses were included with the random sampling method. The inclusion criteria of the participants in the study were as follows: those aged 20 years and over, those without diagnosed any musculoskeletal and mental disorders. Nurses were visited by researchers in their clinics and data were collected via a questionnaire form. In the questionnaire form, socio-demographic characteristics of the nurses were questioned. Age, gender, graduation, marital status, seniority, types of working and clinic, chronic condition were socio-demographic variables. Also to evaluate MSD “Cornell musculoskeletal discomfort questionnaire” and to determine the severity of insomnia “insomnia severity index” were used. Survey forms took an average of 30 minutes to respond.

### Cornell musculoskeletal discomfort questionnaire

The Cornell musculoskeletal discomfort questionnaire (CMDQ) is a data collection tool developed in the Human Factors and Ergonomics Laboratory at Cornell University for the assessment of musculoskeletal symptoms among the English-speaking workforce^17^. It determines the frequency and severity of pain or discomfort and assesses its impact on business performance in 11 different body regions (neck, shoulder, back, upper arm, waist, forearm, wrist, hip, upper leg, knee, and lower leg) during the last week. The frequency of pain or discomfort was rated between never (0) and many times every day (4); severity was rated between mild (1) to very severe (3), and its impact on job performance was rated never obstacle (1) to very obstacle (3).

The questionnaire consisted of right and left subcategories of other extremity sections except neck, back, waist, and hip region. Weighted scores is calculated from the areas of frequency, severity, and work performance for each body region. The weighted score for each body region takes a value between 0 and 90. The increased scores shows that effect on pain frequency, severity, and job performance^17^. The Turkish validity and reliability study was conducted by Erdinc et al., in 2011^18^ and the Cronbach alpha value was 0.876 for frequency, 0.895 for severity, and 0.875 for work performance.

In this study right shoulder, right upper arm, right forearm, and right wrist considered as right upper extremity and left shoulder, left upper arm, left forearm, and left wrist were considered as left upper extremity. Also, right upper leg, right knee, right lower leg were evaluated as lower right extremity and left upper leg, left knee, and lower left leg were considered as lower left extremity. The weighted score is between 0-360 on the right and left upper extremities and 0-270 on the right and left lower extremities.

### Insomnia severity index

This index, which was developed to determine the degree of insomnia symptoms, can be used in normal community screening and clinical evaluation of insomnia. It is a five-point Likert-type scale consisting of seven items. Each item is scored between 0 and 4, and the total score ranges from 0 to 28. The scores of 0-7 indicates clinically insignificant insomnia, 8-14 shows insomnia lower threshold, 15-21 shows clinical insomnia (moderate severity), 22-28 shows clinical insomnia (severe)^19^. The Turkish validity and reliability study was conducted by Boysan et al., in 2010^20^. The internal consistency coefficient of the scale was found to be 0.79^20^.

### Ethical consent

This study was planned in accordance with the Helsinki principles and consent was obtained from the local ethics committee. After giving information about the study, informed consent was obtained from the nurses to participate in the study.

### Statistical analysis

All data were analyzed via using SPSS 22.0 program. In analysis, percentage, average, independent t-test and chi-square test was used to compare categorical variables. Also, multiple binary logistic regression analyses were used. To predict significant factors for insomnia, odds ratio (OR) and 95% confidence interval (CI) were calculated. In evaluations, *p*<0.05 value considered as statistically significant.

## RESULTS

In the study group, 66.6% were aged ≤35 years with the mean age 31.8±8.4 years, 77.8% were women, 83.3% were university graduates, and 57.7% were married. The rate of nurses those with <10 years in their profession was 55.6% while 81.6% were working with shifts and 56.3% were working in internal clinics.

The average scores of the participants obtained from the Cornell musculoskeletal disorder questionnaire and insomnia severity index were shown in Table 1. Accordingly, the body area where the participants experience the most MSD was the waist.

**Table 1.** The distribution of the scores obtained from the scales.

Scales	X±SS	Minimum-maximum values that can be obtained from the scale
**Cornell musculoskeletal disorders questionnaire**		
Neck	6,89±5,32	0-90
Back	7,22±5,60	0-90
Waist	7,86±5,56	0-90
Hip	4,57±4,73	0-90
Upper extremity		
Right	18,07±14,92	0-360
Left	15,32±13,76	0-360
Lower extremity		
Right	23,76±17,90	0-270
Left	21,76±17,99	0-270
**Insomnia severity index**	10,92±5,62	0-28

Musculoskeletal disorder in lower extremities was higher than upper extremities. In addition, there were more MSD on the right side of the body than on the left side. The severity of insomnia among the participants was at the threshold level.

**Figure 1.**
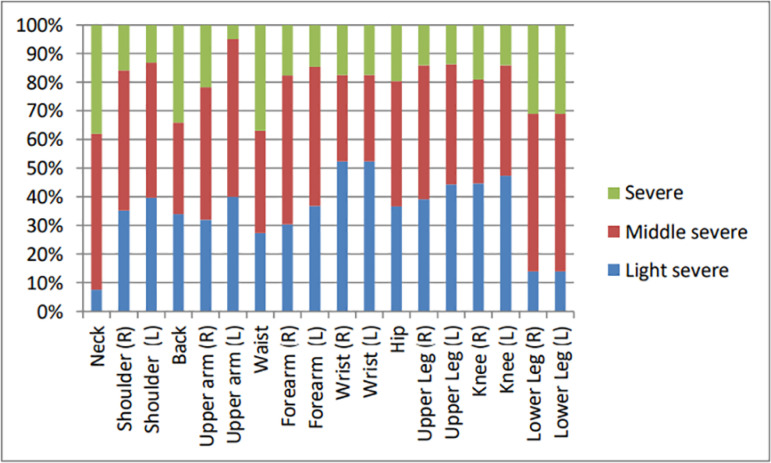
The degree of pain or discomfort according to body areas.

The pain and discomfort experienced by the participants according to their body areas were shown in Table 2. Body areas where the participants had the most pain complaints and had most obstacles in their job were waist, neck, and back. The severity of the discomfort was mostly moderate in painful areas. The areas with the most severe pain were observed to be the neck, back, and waist (Figure 1). According to some characteristics of the participants, the average scores of the Cornell musculoskeletal disorders questionnaire were presented in Table 3.

**Table 2.** Existence of musculoskeletal disorders and obstacle to doing job according to body areas.

Body areas	Musculoskeletal disorder		Obstacle status for job	
	Had n (%)	Had not n (%)	Obstacle n (%)	Not obstacle n (%)
**Neck Shoulder**	198 (67,6)	95 (32,4)	107 (36,6)	186 (63,4)
Right	166 (56,7)	127 (43,3)	92 (31,4)	201 (68,6)
Left	137 (46,8)	156 (53,2)	72 (24,6)	221 (75,4)
Back	198 (67,6)	95 (32,4)	114 (38,9)	179 (61,1)
**Upper arm**				
Right	115 (39,2)	178 (60,8)	50 (17,1)	243 (82,9)
Left	93 (31,7)	200 (68,3)	37 (12,7)	256 (87,3)
**Waist Forearm**	214 (73,0)	79 (27,0)	132 (45,1)	161 (54,9)
Right	90 (30,7)	203 (69,3)	37 (12,6)	256 (87,4)
Left	71 (24,2)	222 (75,8)	31 (10,6)	262 (89,4)
**Wrist**				
Right	93 (31,7)	200 (68,3)	45 (15,4)	248 (84,6)
Left	79 (27,0)	214 (73,0)	37 (12,7)	256 (87,3)
**Hip Upper leg**	**121 (41,3)**	**172 (58,7)**	**63 (21,5)**	**230 (78,5)**
Right	137 (46,8)	156 (53,2)	72 (24,6)	221 (75,4)
Left	124 (42,3)	169 (57,7)	62 (21,1)	231 (78,9)
**Knee**				
Right	154 (52,6)	139 (47,4)	84 (28,7)	209 (71,3)
Left	135 (46,1)	158 (53,9)	74 (25,2)	219 (74,8)
**Lower leg**				
Right	160 (54,6)	133 (45,4)	88 (30,0)	205 (70,0)
Left	148 (50,5)	145 (49,5)	76 (25,9)	217 (74,1)

**Table 3.** Scores obtained from the Cornell musculoskeletal disorders questionnaire according to the socio- demographic characteristics of the participants.

Characteristics	Neck	Back	Waist	Hip	Upper extremity (R)	Upper extremity (L)	Lower extremity (R)	Lower extremity (L)
**Age groups (year)**								
≤35	6,85±5,28	7,33±5,53	7,59±5,29	4,37±4,59	17,07±13,18	14,84±12,49	23,34±17,22	21,66±17,63
>35	6,96±5,42	7,00±5,75	8,38±6,05	4,97±5,00	20,09±17,82	16,27±16,01	24,58±19,26	21,97±18,77
*p*	0,927	0,450	0,382	0,313	0,606	0,850	0,974	0,767
**Gender**								
Female	7,31±5,49	7,45±5,66	8,15±5,55	4,90±4,98	19,11±15,85	15,94±14,74	24,88±18,55	22,57±18,68
male	5,42±4,37	6,40±5,33	6,83±5,52	3,42±3,54	14,40±10,27	13,13±9,32	19,81±14,87	18,94±15,09
*p*	**0,016**	0,159	0,068	**0,047**	**0,039**	0,430	0,071	0,404
**Graduation**								
High school	6,93±5,25	6,35±5,14	7,76±5,43	4,34±4,22	13,95±10,84	12,07±9,59	22,45±16,25	
University	6,88±5,34	7,39±5,68	7,87±5,59	4,61±4,84	18,90±15,50	15,97±14,38	24,02±18,24	
*p*	0,085	0,291	0,931	0,866	**0,026**	0,088	0,770	0,688
**Marital status**								
Married	7,12±5,20	7,57±5,73	8,25±5,69	4,79±4,74	18,94±15,48	15,94±14,13	25,64±18,43	23,13±19,02
Non-married	6,58±5,47	6,75±5,40	7,31±5,35	4,27±4,73	16,90±14,11	14,47±13,25	21,18±16,90	19,89±16,37
p*	0,213	0,246	0,133	0,145	0,125	0,294	0,027	0,216
**Seniority (year)**								
<10	6,80±5,26	7,26±5,47	7,30±5,04	4,04±4,20	16,48±12,39	14,47±11,24	22,69±15,78	21,35±16,24
≥10	7,00±5,40	7,17±5,77	8,55±6,10	5,23±5,27	20,08±17,46	16,39±16,36	25,10±20,23	22,27±20,01
*p*	0,741	0,895	0,056	**0,033**	**0,041**	0,236	0,252	0,665
**Types of working**								
Daytime	7,97±5,72	7,87±5,65	7,52±5,46	5,34±6,16	17,63±14,08	13,17±11,31	24,50±16,80	19,04±15,24
Shift	6,64±5,20	7,07±5,58	9,34±5,79	4,39±4,34	18,17±15,13	15,80±14,23	23,59±18,17	22,38±18,53
*p*	0,117	0,293	**0,028**	0,924	0,818	0,289	0,445	0,282
**Types of clinic**								
Internal	7,08±5,52	7,27±5,82	7,80±5,71	4,29±4,84	19,12±16,57	15,86±14,67	24,18±18,95	22,02±18,86
Surgical	6,64±5,06	7,15±5,31	7,93±5,38	4,93±4,59	16,71±12,39	14,63±12,50	23,21±16,51	21,43±16,86
*p*	0,312	0,114	0,180	0,875	**0,021**	0,280	0,079	0,117
**Chronic condition**								
Had	7,94±5,20	8,52±5,52	8,85±6,29	5,54±5,45	19,21±15,16	16,12±14,03	28,08±22,05	25,82±21,70
Had not	6,53±5,32	6,78±5,56	7,52±5,26	4,24±4,43	17,69±14,86	15,05±13,69	22,29±16,06	20,39±16,38
*p*	**0,018**	**0,011**	0,166	0,072	0,383	0,497	0,155	0,167
**BMI (kg/m2)**								
<25	7,09±5,35	7,28±5,54	7,52±5,28	4,35±4,39	17,60±14,27	15,41±13,46	22,83±17,19	20,77±17,51
≥ 25	6,52±5,26	7,11±5,72	8,47±6,01	4,96±5,30	18,93±16,09	15,15±14,35	25,44±19,11	23,56±18,78
*p*	0,279	0,622	0,278	0,614	0,766	0,520	0,303	0,148

There was no statistically significant difference between the mean scores of age and BMI with MSD scores (*p*>0.05). In our study, it was determined that female nurses had significantly higher scores in neck, hips, upper right extremity than male nurses (*p*<0.05).

Nurses those with university graduates had higher scores in the upper right extremity compared to high school graduates (*p*<0.05). It was determined that the scores obtained from the lower right extremity were higher than the married people (*p*<0.05). Married nurses had higher scores in lower right extremity than unmarried (*p*<0.05). The scores obtained from hip and upper right extremity were higher among nurses those with ≥10 years seniority (*p*<0.05). The scores obtained from the waist were found to be significantly higher in those with shift working compared to those with daytime working. Nurses with chronic condition had significantly higher scores in neck and back areas than those without chronic condition (*p*<0.05).

The severity of insomnia according to the body regions were shown in Table 4. It was determined that the threshold level of insomnia was significantly higher in those with MSD except the knee and lower leg region (*p*<0.01, *p*<0.05). Multiple binary logistic regression analysis of painful body areas affecting insomnia was given in Table 5. Insomnia was 1.21 (aOR=1.21; 95% CI=1.31-2.45; *p*=0.000) times higher in those with neck pain, 1.04 (aOR=1.04; 95% CI=1.02-1.98; *p*=0.023) times higher in those with right shoulder pain and 1.12 (aOR=1.12; 95% CI=1.06-2.12; *p*=0.000) times higher in those with right upper arm pain.

**Table 4.** The severity of insomnia according to painful body areas in the participants.

	Insomnia								
Painful body areas	Insignificant (n=89)		Threshold (n=127)		Middle/severe (n=77)		Total (n=293)		χ^2^/p
	No.	%	No.	%	No.	%	No.	%	
Neck	43	21,7	88	44,5	67	33,8	198	67,6	21,646/0,000
Shoulder (R)	36	21,7	76	45,8	54	32,5	166	56,7	13,671/0,000
Shoulder (L)	31	22,6	62	45,3	44	32,1	137	46,8	7,303/0,013
Back	49	24,7	94	47,5	55	27,8	198	67,6	9,146/0,002
Upper arm (R)	22	19,1	51	44,4	42	36,5	115	39,2	11,318/0,000
Upper arm (L)	20	21,5	42	45,2	31	33,3	93	31,7	5,069/0,024
Waist	53	24,8	101	47,2	60	28,0	214	73,0	11,807/0,003
Forearm (R)	17	18,3	43	48,3	30	33,4	90	30,7	11,250/0,004
Wrist (R)	20	21,5	39	41,9	34	36,6	93	31,7	5,069/0,024
Hip	23	19,0	64	52,9	34	28,1	121	41,3	12,593/0,000
Upper leg (R)	32	23,4	63	46,0	42	30,6	137	46,8	5,992/0,014

**Table 5.** Multiple logistic regression analysis of painful body areas affecting insomnia.

Variables	Adjusted OR	95% CI	p
Neck pain	1,21	1.31-2.45	0.000
Shoulder (R) pain	1.04	1.02-1.98	0,023
Upper arm (R) pain	1.12	1.06-2.02	0,000

## DISCUSSION

This study was conducted with the considering that MSD were an important job stress among nurses and may affect the severity of insomnia. Nursing profession requires physical difficulties such as weight lifting, improper body posture, and exposure to psychological stress factors. Continuous exposure to these risk factors causes MSD^21^.

In the current study, it was found that the body regions with the most pain complaints were waist (73%), neck (67.6%), back (67.6%), and shoulder (right 56.7%). Also, these regions were obstacle for job performance (Table 2). A study conducted in USA reported that the body areas where the MSD were seen most frequently among the nurses were waist, back, neck, and shoulders^22^. Another study has shown that pain and discomfort were more common in the back region among Jordanian nurses^23^.

Similar to USA and Jordanian studies, in Turkey MSD were shown in waist, back, neck, shoulders, and lower extremity areas among nurses^24,25^. The result obtained from our study was compatible with the results of both national and international studies. Nursing activities are a dynamic process in patient care and treatment. In this process, physical movements may not suitable for the body posture that cause difficulties in the musculoskeletal structure.

Some groups (women, those with higher seniority, shift workers, those with major chronic diseases such as diabetes) were shown to be more prone to MSD among nursing profession^26,27,28^. Indeed, similar results were found in this study. Female nurses and nurses those with higher educated, married, higher seniority, shift working, working in internal clinics, and chronic diseases were more pain complaints in any body area (Table 3). Frequent occurrence of MSD in female nurses can be affected by the traditional gender roles and physiological processes (pregnancy, birth, etc.).

There is evidence that education is an important risk factor for the formation of MSDs and that highly educated individual’s work in better positions in organizations^29,30,31,32^. However, in accordance with our study, it was reported that MSDs were more frequent among highly educated nurses^33,34^. Such a result may be due to highly educated nurses had more intense work and responsibilities in clinics.

Marriage brings additional tasks and responsibilities to individual’s life. This increased duties and responsibilities may cause nurses to experience more musculoskeletal pain. Also higher seniority in profession can be a pressure on musculoskeletal system and cause pain.

In the present study, the rates of MSDs in different parts of the body were found to be greater in those who had a chronic illness. This is an expected result. It was reported that long working hours cause both physical and psycho-social problems and increase MSDs^13,35^. Work-related MSD not only affect work productivity, but also negatively affect quality of life^36,37^. It was stated that pain in the musculoskeletal system impairs sleep patterns^38^. Musculoskeletal pain is a known cause of sleep disturbance^39^. All these evidence supported our findings. In our study, insomnia severity was at the threshold level in all group. The severity of insomnia at the threshold level was found to be significantly higher in nurses with pain complaints in almost all body areas except knee and lower leg region (Table 4). Neck pain, right shoulder pain, and right upper arm pain were risk factors for insomnia (Table 5).

## CONCLUSION

In our study, it was determined that MSD were common in the upper body areas and the severity of insomnia was at the threshold level. Neck, right shoulder, and right upper arm were risky body areas for insomnia. All these problems can trigger other physical and mental disorders and reduce job performance. Nurses should be trained on ergonomics and encouraged for exercises to reduce the frequency of musculoskeletal disorders.
